# Transcriptional Responses of In Vitro Blood–Brain Barrier Models to Shear Stress

**DOI:** 10.3390/biom15020193

**Published:** 2025-01-29

**Authors:** Koji L. Foreman, Benjamin D. Gastfriend, Moriah E. Katt, Sean P. Palecek, Eric V. Shusta

**Affiliations:** 1 Department of Chemical and Biological Engineering, University of Wisconsin-Madison, Madison, WI 53706, USA; foreman03@gmail.com (K.L.F.); bgastfriend@health.ucsd.edu (B.D.G.); moriah.katt@mail.wvu.edu (M.E.K.); 2 Department of Neurosurgery, University of Wisconsin-Madison, Madison, WI 53792, USA

**Keywords:** human pluripotent stem cell, blood–brain barrier, shear stress, fluid flow, brain endothelium, transcriptomics

## Abstract

Endothelial cells throughout the body sense blood flow, eliciting transcriptional and phenotypic responses. The brain endothelium, known as the blood–brain barrier (BBB), possesses unique barrier and transport properties, which are in part regulated by blood flow. We utilized RNA sequencing to analyze the transcriptome of primary cultured rat brain microvascular endothelial cells (BMECs), as well as three human induced pluripotent stem cell-derived models. We compared the transcriptional responses of these cells to either low (0.5 dyne/cm^2^) or high (12 dyne/cm^2^) shear stresses, and subsequent analyses identified genes and pathways that were influenced by shear including key BBB-associated genes (*SLC2A1*, *LSR*, *PLVAP*) and canonical endothelial shear-stress-response transcription factors (*KLF2*, *KLF4*). In addition, our analysis suggests that shear alone is insufficient to rescue the de-differentiation caused by in vitro primary BMEC culture. Overall, these datasets and analyses provide new insights into the influence of shear on BBB models that will aid in model selection and guide further model development.

## 1. Introduction

Despite the prevalence of central nervous system (CNS) diseases, new therapeutic treatments are approved at a much lower rate than therapies targeting other tissues [1,2]. A major challenge to treating disorders within the CNS is the general inaccessibility of circulating drugs to brain tissue, driven in part by the blood–brain barrier (BBB) [1,2,3]. The brain endothelial cells comprising the BBB mediate selective resistance to molecular transport. Brain microvascular endothelial cells (BMECs), working in conjunction with the surrounding pericytes, astrocytes, and neurons of the neurovascular unit (NVU), help to maintain brain homeostasis by facilitating the transport of nutrients, including iron and glucose, while simultaneously insulating the brain from changes in the blood composition, such as the brain accumulation of blood-borne drugs [4,5,6]. While the brain-specific properties of BMECs are largely thought to arise from NVU interactions, the CNS endothelium also experiences physical cues, such as the shear stress originating from blood flow. Blood flow has long been known to impact the behavior of aortic [7,8], capillary [9], and human umbilical vein [10,11] endothelial cells. As a few examples of the effects of flow and fluid shear stress, endothelial cells undergo structural changes and align [7,8,10], have reduced proliferation [12,13], exhibit downregulated p21 signaling [14] and upregulated TGFβ [15,16] and JAK-STAT [17,18] signaling, and possess altered transcriptomes [19,20] that include the increased expression of transcription factors such as *KLF2* [21] and *KLF4* [22,23].

The transcriptional response of the brain endothelium in response to shear has not been studied as extensively, particularly in human cells. However, numerous human BBB models ranging from immortalized BMECs to human hematopoietic and human pluripotent stem cell (hPSC)-derived models have previously been generated [24,25]. Of particular interest to this study are induced pluripotent stem cell (iPSC)-derived BBB models, which can facilitate human disease modeling [26,27,28], as well as provide an isogenic source of other cell types of the NVU [29]. Studies using hematopoietic stem cell- and hPSC-derived BBB models have noted transcriptional increases in many BBB-relevant genes under shear stress [30,31], suggesting a BBB-specific response to shear stress. For example, genes encoding efflux transporters, including P-gp (*ABCB1*), tight junction forming proteins like claudin-5 (*CLDN5*), and the canonical BBB glucose transporter GLUT1 (*SLC2A1*), were upregulated by shear in these models. However, other work has suggested that the general shear response phenotype is less pronounced in in vitro BBB models compared to endothelial cells cultured from other tissues [10,11,32]. For example, both an hPSC BBB model and immortalized human BMEC model reported a lack of cell alignment under flow, a well characterized response of endothelial cells to shear stress [10,11], although there was a reduction in both apoptosis and proliferation [11], consistent with non-brain endothelial cells [13]. Unfortunately, direct comparisons between various published datasets can be difficult due to differences in the shear magnitude, duration of shear, parenchymal cell types, vascular model, transcriptomic sequencing depth, in vitro chip geometry, and data availability.

Here, to partially address this knowledge gap related to how shear affects in vitro models of the BBB, we employed RNA sequencing to directly compare the shear responses of four endothelial or BBB models: primary rat BMECs (rBMECs), human iPSC-derived ECs without a BBB character (hECs) [33], Wnt-activated iPSC-derived ECs with a CNS-like character (hCECs) [34,35], and iPSC-derived brain microvascular endothelial cell-like (hBMEC-like) cells [36]. Each model was subjected to the same magnitudes of low (~0.5 dyne/cm^2^) and physiological (~12 dyne/cm^2^) shear stress for 72 h [10,11,37] in identical microfluidic chips to enable a direct comparison of their transcriptional responses to shear flow. A subsequent transcriptional analysis and comparison were performed to identify genes and pathways that are shear-responsive in each model.

## 2. Materials and Methods

### 2.1. iPSC Culture

IMR90-4 human iPSCs (WiCell Research Institute, Madison, WI, USA) were maintained in E8 medium (Stem Cell Technologies, Vancouver, BC, Canada) on Matrigel (WiCell Research Institute, Madison, WI, USA) -coated tissue culture treated polystyrene six-well plates (Corning, Waltham, MA, USA). At 50–70% confluence, iPSCs were passaged at 1:6 using Versene (Gibco, Waltham, MA, USA).

### 2.2. iPSC-EPC Differentiation (hEPCs)

Human endothelial progenitor cells (hEPCs) were differentiated from iPSCs as previously described [33,34]. Briefly, when iPSCs reached confluence, they were detached using 1 mL of Accutase (Stem Cell Technologies, Vancouver, BC, Canada) per well for 7–8 min. When most cells were visibly suspended, the cell solution was triturated to break up clumps before the addition of 4 mL of DMEM/F12 (Gibco, Waltham, MA, USA) per mL of cell solution. Cells were centrifuged at 200 RCF for 5 min, and the supernatant aspirated. The cell pellet was resuspended in E8 (Gibco, Waltham, MA, USA) + 10 µM Y-27632 (Tocris Bioscience, Bristol, UK). Twelve-well Matrigel (WiCell, Madison, WI, USA) coated tissue culture treated polystyrene plates (Corning, Waltham, MA, USA) were seeded with 70,000 cells/well in 1 mL E8 (Gibco, Waltham, MA, USA) + 10 µM Y-27632 per well. Cells were fed 1 mL of E8 (Gibco, Waltham, MA, USA) per well for two days. Afterwards cells were fed 2 mL of LaSR (500 mL of Advanced DMEM/F12 (Gibco, Waltham, MA, USA), 30 mg ascorbic acid (Thermofisher, Waltham, MA, USA), 6.25 mL of GlutaMAX (Gibco, Waltham, MA, USA)) + 8 µM CHIR99021 (Tocris Bioscience, Bristol, UK) per well daily for two days. Cells were then fed 2 mL of LaSR per well daily for an additional 3 days. Cells were then lifted and sorted via magnetic activated cell sorting (MACS) using the Stem Cell Technologies EasySep Human FITC Positive Selection Kit II (Gibco, Waltham, MA, USA), using either the CD34-FITC (Miltenyi Biotec, Charlestown, MA, USA) or CD31-FITC (BD Biosciences, Franklin Lakes, NJ, USA) antibodies for positive selection. Cells were resuspended in HECSR (HESFM (Gibco, Waltham, MA, USA) + B-27 (Gibco, Waltham, MA, USA)) + 10% DMSO (Sigma-Aldrich, St. Louis, MO, USA) + 30% FBS (Gibco, Waltham, MA, USA) at ~5 million cells/cm^2^; 1 mL vials (Thermofisher Scientific, Waltham, MA, USA) were then frozen in a freezing container (ULAB, Memphis, TN, USA) following the manufacturers’ instructions at −80 °C for at least overnight before being banked in liquid nitrogen for long-term storage.

### 2.3. hCEC and hEC Differentiation

**Figure 1:**
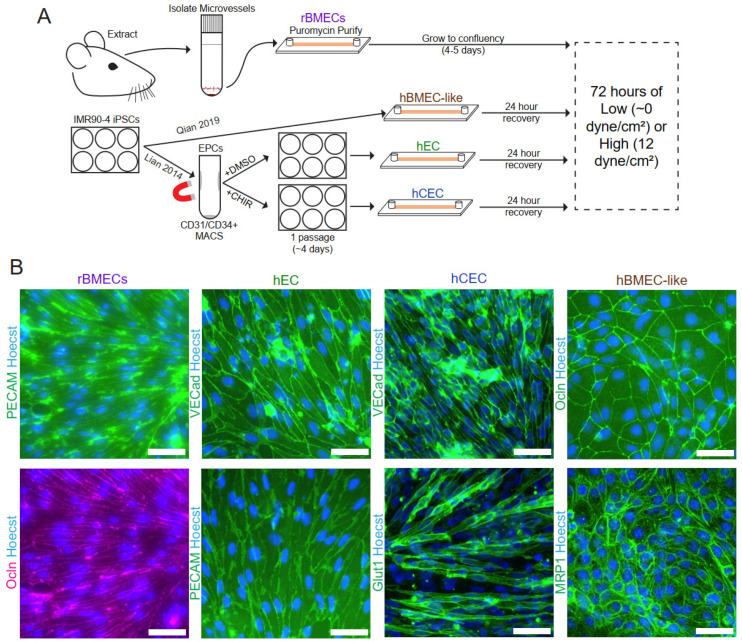
Model establishment and validation. (**A**) Schematic of model isolation/differentiation methods prior to the application of shear stress. (**B**) Immunofluorescent analysis of endothelial and BBB markers in rBMECs, hECs, hCECs, and hBMEC-like cells with protein expression and localization consistent with the previously published literature for these models. rBMEC immunofluorescence panels are from the same microscope field of a sample double-labeled for PECAM-1 and Occludin. Scale bars = 50 μm.

iPSC differentiation to human CNS-like endothelial cells (hCECs) and human endothelial cells (hECs) was adopted from previously published protocols [34,35] (Figure S1). Briefly, hEPCs were thawed into HECSR + 20 ng/mL FGF2 (WiCell Research Institute, Madison, WI, USA) + 10 ng/mL VEGF (Tocris Bioscience, Bristol, UK) + 4 µM CHIR99021 for hCECs or DMSO for hECs. Media were changed every 48 h until cells reached confluency. At confluency, cells were passaged from 1 well of a 6-well plate to 2 µSlide|Luer microfluidic chips (ibidiUSA, Fitchburg, WI, USA). Seeded chips were then allowed to settle for a minimum of 4 h prior to the application of <1 mL/min fluid flow for 24 h to ensure confluency was reached before shear application, using HECSR + 20 ng/mL FGF2 + 10 ng/mL VEGF +1x Antibiotic–Antimycotic (Gibco, Waltham, MA, USA) + 4 µM CHIR99021 for hCECs or DMSO for hECs. Of note, the differentiation of hECs and hCECs can generate a subpopulation of smooth muscle-like cells over time [33], but very few of these cells were observed in this study due to passaging, as well as the addition of CHIR99021 to the hCEC cultures (Figure 1B) [33,34,35].

### 2.4. hBMEC-like Cell Differentiation

hBMEC-like cells were differentiated from iPSCs as previously described [36] (Figure S1). iPSCs were seeded at 35,000 cells/cm^2^ into Matrigel-coated tissue culture treated 6-well plates in 2 mL of E8 medium + 10 µM Y-27632. Cells were then fed 2 mL of E8 for two days before switching to DMEM/F12 + 1x Non-essential Amino Acids (NEAA, Gibco, Waltham, MA, USA) + 0.5x GlutaMAX (Gibco, Waltham, MA, USA) + 0.1 mM β-mercaptoethanol (Thermofisher Scientific, Waltham, MA, USA) (DeSR1) + 4 µM CHIR99021; 24 h later, media were replaced with DMEM/F12 + 1x NEAA + 0.5x GlutaMAX + 0.1 mM β-mercaptoethanol + 1x B27 (DeSR2) and maintained with media changes for 5 days. Cells were then fed HESFM (Gibco) + 1x B27 (HECSR) + 10 µM retinoic acid (Sigma-Aldrich, St. Louis, MO) + 20 ng/mL FGF2. After 48 h, cells were replated onto collagen IV (Sigma-Aldrich, St. Louis, MO) and fibronectin (Gibco, Waltham, MA, USA) -coated ibidi microfluidic chips. Cells were maintained under minimal flow for 24 h in HESFM + 1x B27 + 10 µM retinoic acid + 20 ng/mL FGF2 + 1x Antibiotic–Antimycotic (Gibco, Waltham, MA, USA) before changing to HECSR + 1x Antibiotic–Antimycotic (ThermoFisher) and applying shear stress.

### 2.5. rBMEC Purification

Animal experiments were conducted under a protocol approved by the University of Wisconsin–Madison Institutional Animal Care and Use Committee, following NIH guidelines for the care and use of laboratory animals. Endothelial cells isolated from half of a rat brain were seeded into each chip. For experiments where multiple rat brains were needed, brains were pooled and treated as a single isolation. Brains from male Sprague–Dawley rats between 220 and 250 g were isolated, and brain hemispheres were stored in DMEM on ice while awaiting processing. Microvessels were isolated as previously described [38]. Briefly, after rolling brain hemispheres on KimWipes (Fisher Scientific, Waltham, MA, USA) to capture meninges, cortices were dissociated through vigorous chopping and then added to 1 mL of DMEM. This brain solution was then transferred to a 50 mL conical (Thermo Fisher Scientific, Waltham, MA, USA), and an additional 9 mL of DMEM added. The solution was triturated to mechanically disassociate fragments before the addition of 0.75 mL of 10 mg/mL collagenase (Sigma-Aldrich, St. Louis, MO, USA) and 150 µL of 1 mg/mL DNase I (Qiagen, Hilden, Germany). The solution was incubated for 75 min at 37 °C on a shaker at 250 rpm. During the incubation, a Percoll gradient was prepared in a 30 mL Oakridge tube (Thermofisher Scientific, Waltham, MA, USA) with 10 mL of Percoll (Sigma-Aldrich, St. Louis, MO, USA), 19 mL of PBS (Gibco, Waltham, MA, USA), 1 mL of 10x PBS (Sigma-Aldrich, St. Louis, MO, USA), and 1 mL of FBS and centrifuged at 30,000 RCF for 30 min. After incubation, the brain homogenate was diluted to 40 mL in DMEM and then centrifuged at 1000 RCF for 8 min, and the supernatant was aspirated. The microvessel pellet was resuspended in 25 mL of 20% (*m*/*v*) BSA in DMEM and triturated to further mechanically separate the vessels from the brain cells. The mixture was then centrifuged at 1000 RCF and 4 °C for 20 min to separate the bulk of the neurons and glia from the microvessel pellet. The neuron/glia layer and supernatant were aspirated before resuspension and trituration in 6.75 mL of DMEM. Then, 0.5 mL of 10 mg/mL collagenase–dispase (Sigma-Aldrich, St. Louis, MO, USA) and 50 µL of 1 mg/mL DNase 1 were added to the microvessel pellet, and the solution was incubated for 1 h at 37 °C on a shaker at 250 RPM. Microvessel fragments were centrifuged at 700 RCF for 6 min, and the supernatant was aspirated. The pellet was resuspended in 1 mL of DMEM and added to the prepared Percoll gradient and centrifuged at 1000 RCF for 10 min at 4 °C. After the gradient, a 5 mL syringe (BD, Franklin Lakes, NJ, USA) with a blunt 16 gauge 5″ needle (BD, Franklin Lakes, NJ, USA) was used to extract the microvessel-containing layer, which was diluted with DMEM at 4 mL per mL of microvessel solution. The microvessel solution was centrifuged at 200 RCF for 10 min and then resuspended in 200 uL of rat primary medium (DMEM + 10% FBS + 1x GlutaMAX + 1x Antibiotic–Antimycotic (ThermoFisher) + 4 µg/mL puromycin (Sigma-Aldrich, St. Louis, MO, USA) + 100 ng/mL heparin (Sigma-Aldrich, St. Louis, MO, USA) + 20 ng/mL FGF2) and plated into ibidi microfluidic chips coated with 250–300 µL of 0.2 mg/mL collagen IV and 0.2 mg/mL fibronectin for 1 h. Every 8 h, chips were fed with 200 uL of rat primary medium until confluent (usually between 3–4 days) before proceeding with connection to microfluidic devices. After connection to the microfluidic pump system, the medium was replaced every 48 h.

### 2.6. Preparation of Microfluidic Devices and Application of Shear Stress

Here, 0.8 mm- and 0.6 mm-height µSlide|Luer chips purchased from ibidi were connected to 1/16″ and 1/32″ internal diameter silicone tubing and nylon components via the female Luer ports on the chips, as shown in Figure S2. Prior to assembly, all non-chip components were washed with water, soap and water, 10% bleach solution, and then ethanol before autoclaving to sterilize them. Shear stress was applied after a confluent monolayer was achieved. Cells exposed to low shear (0.5 dyne/cm^2^) received nutrients and oxygen while matching the geometry of the high-shear condition. During the ramp-up phase in the high-shear chips, shear was increased by 3 dyne/cm^2^ every 30 min until 12 dyne/cm^2^ was reached. Low-shear-stress chips were maintained at 0.5 dyne/cm^2^ over the ramp-up period. Chips were then maintained under flow for 72 h before RNA extraction.

### 2.7. RNA Extraction and Sequencing

Chips were disconnected from the microfluidic device and rinsed with Dulbecco’s Phosphate Buffered Saline with Calcium and Magnesium (DPBS +/+) (Gibco, Waltham, MA, USA). Chips were then assessed under a bright field microscope to ensure that cells remained attached prior to lysing with the cell lysis buffer provided in the Qiagen RNeasy Mini Kit (Qiagen, Hilden, Germany). Samples were then processed using Qiagen Shredder columns and the Qiagen RNeasy Mini Kit. Samples were prepared using polyA enrichment. Library preparation and RNA sequencing were performed by Novogene. Samples were sequenced using paired-end reads.

### 2.8. Immunofluorescent Imaging

For immunofluorescent labeling, cells were initially rinsed twice with cold PBS before fixing with either methanol (Sigma Aldrich, St. Louis, MO, USA) or 4% PFA (Thermofisher Scientific, Waltham, MA, USA) in PBS (Table S1). Afterwards, cells were blocked 10% goat serum (Gibco, Waltham, MA, USA) in DPBS for 1 h at room temperature. Cells were then incubated overnight with the primary antibody (Table S1) at 4 °C while shaking at 30 rpm. Cells were rinsed 3 times with DPBS, and then, secondary antibody solution in 10% goat serum was applied for 1 h at room temperature. Cells were then rinsed twice with PBS and a solution of 2 µg/mL Hoechst 33342 (Sigma Aldrich, St. Louis, MO, USA) in PBS was applied for 10–15 min. The Hoechst solution was aspirated and replaced with PBS. Cells were imaged on a Nikon Eclipse Ti2-E epifluorescence microscope, and images were analyzed with the FIJI package of ImageJ version 1.53e [39].

### 2.9. Statistics and RNA Sequencing Analysis

To process the RNA sequencing files, STAR 2.7.9a via the Github repository was used for alignment to the hg38 genome for human samples and rn7 genome for rat samples [40]. Genome files were provided by the UW Transcriptomics core. featurecounts v2.0.3 was used to generate count tables [41], and differential expression was analyzed using DeSeq2 via BioConductor v3.15 based on the raw counts [42].

Replication was at the differentiation/isolation level, either with replicate cells differentiated from the same iPSC line at different passages or with isolated microvessels taken from replicate rats. Paired DESeq2 was utilized for comparisons between high- and low-shear samples. Statistics reported are those returned via paired DESeq2 analysis. Results for GO were only considered significant if they had an FDR of less than 0.05. GO was performed via the PANTHER online interface [43,44]. GSEA v4.2.3 was run via the application provided by the Broad Institute [45,46]. GSEA gene inputs were ranked based on the negative log base ten of the *p*adj value provided by DESeq2 multiplied by the log_2_ fold change. For any comparison between rats and humans, we utilized Homologene2 from the NCBI dataset, which uses gene names updated to 2019 to assign human orthologs for rat genes. Any gene lacking an ortholog was discarded from the analysis. ChEA3 [47] and BART 2.0 [48] analyses were both run via their respective web portals. For inputs we used only genes with a positive fold change and statistical significance according to DESeq2. For ChEA3, which offers multiple libraries of the aforementioned ChIP-seq meta-analysis, we utilized the “literature” library. Statistical significance was set at an FDR/Irwin-Hall *p*-value (as reported by ChEA3/BART respectively) of less than 0.05. Upstream factors that did not have an average TPM of 1 or greater were discarded as non-expressed.

## 3. Results

### 3.1. Validation of Endothelial Cell Isolation and Differentiation

Rat brain microvessels (Figure 1A) were isolated using standard enzymatic isolation and culture techniques with outgrowing nonendothelial contaminant cells removed via puromycin treatment as previously described [38,49]. Importantly, the microvessels were directly plated into the microfluidic device to minimize the time ex vivo, which is known to drive the de-differentiation of rBMECs [50]. Rat BMECs cultured in this way have been shown to express efflux transporters, as well as numerous endothelial and BBB markers [38], and rBMECs grown in companion 96-well static tissue culture wells were validated for PECAM-1 and occludin expression and localization (Figure 1B).

All iPSC-derived models were differentiated from the IMR90-4 iPSC line. For the iPSC-derived endothelial-like cells (hECs) and the Wnt-activated CNS-like iPSC-derived EC (hCEC) models, iPSCs were differentiated according to previously published protocols [33,34,35]. hECs represent a naïve EC lacking organospecific signatures [34,35] and served as both a generic EC control for the shear response and provided a genetically matched comparison to the hCECs and hBMEC-like cells derived from the same iPSC line. iPSC-derived endothelial progenitor-like cells (hEPCs) were first generated from iPSCs according to previously published protocols [33] before enrichment via magnetic activated cell sorting (MACS). The hEPC population was then either maintained for one passage to yield hECs or treated with CHIR99021, a GSK3 inhibitor, to generate hCECs (Figure 1A and Figure S1). The hECs and hCECs expressed PECAM-1 and VE-cadherin as expected [33,34] (Figure 1B). hCECs also display several hallmarks of in vivo BMECs, including elevated GLUT1 expression, decreased PLVAP expression, and increased barrier properties compared to hECs [34]. Here, we demonstrated GLUT-1 and VE-cadherin expression in hCECs [34] (Figure 1B). For the fourth model, iPSC-derived brain microvascular endothelial cell-like (hBMEC-like) cells were differentiated as described previously [36]. The hBMEC-like cells have substantial barrier and transport properties and the expression of barrier-related genes (e.g., occludin and MRP1, Figure 1B) but express epithelial-related genes and significantly lower levels of EC genes than the other three models used here [34,36,51,52,53,54].

### 3.2. Shear Stress Drives a Transcriptional Shift in Each In Vitro Endothelial and BBB Model

All models were seeded onto ibidi microfluidic chips (Figure S2) and allowed to reach confluence before the application of fluid flow (Figure 1A). Flow rates were chosen to generate shear stress at two levels: low shear stress at ~0.5 dyne/cm^2^ which allows for nutrient supply to the cells in the chip but which is below where shear responses are typically detected in endothelial cells [10,11,25], and high shear stress of 12 dyne/cm^2^, which is approximately the physiological level observed in the brain microvasculature [7,55]. Flow was applied for 72 h before cells were lysed and collected for RNA-sequencing analysis. RNA quality measures were generated via MultiQC [56], and the general summary is available in Supplemental File S1, and all transcript counts are provided in Supplemental File S2.

**Figure 2:**
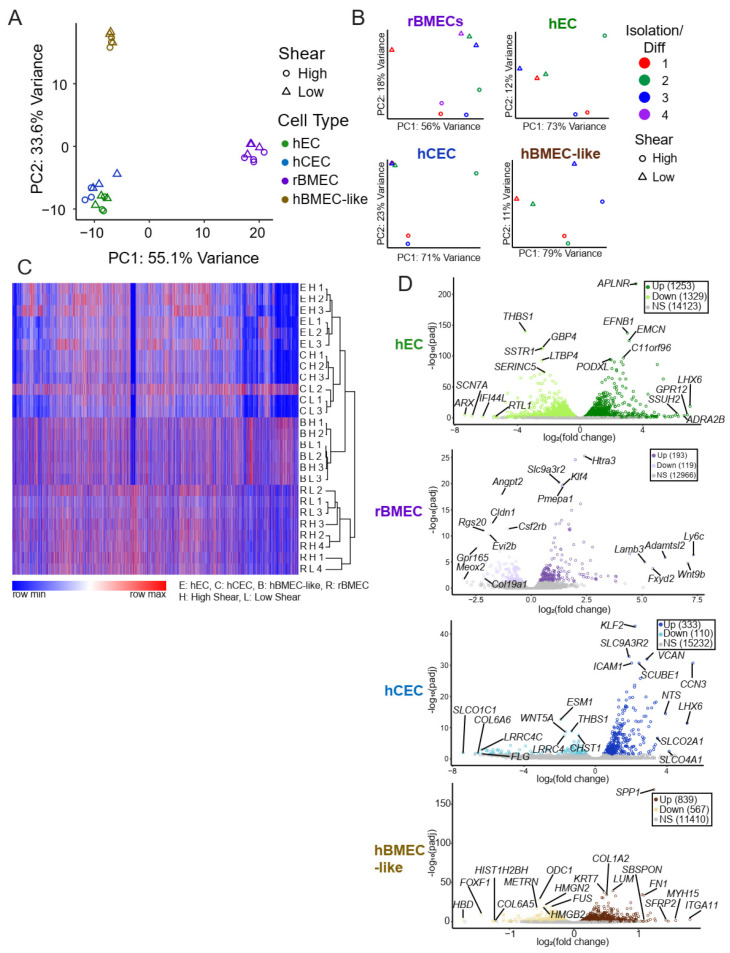
Unbiased whole transcriptomic analysis. (**A**) Principal component analysis utilizing TPM for each model under low and high shear only using genes that have an established rat orthologue. (**B**) PCA of each individual model utilizing variance-stabilized counts according to DESeq2. Rat brain samples were collected over three separate rat brain isolations and underwent shear in three separate events and were analyzed via paired analysis both for PCA and DESeq2s. Similarly, three separate iPSC differentiations were collected with and without shear. (**C**) Overall heat map of hierarchical clustering of log_10_(TPM + 1) from all models. Any genes without an established ortholog or with an average TPM across all samples of less than 1 were excluded. We observed an approximately 10% loss of genes due to the lack of orthologs. Gene clustering is from hECs, and order is maintained across all other samples. The color indicates the row-normalized minimum or maximum per gene. Heat map and hierarchical clustering were performed using the Morpheus tool from the Broad Institute (https://software.broadinstitute.org/morpheus, accessed on 18 December 2024). (**D**) Volcano plots for each model with the top five genes with the highest fold change and the five with the lowest *p*adj are labeled. Grey indicates statistically insignificant fold changes (*p*adj > 0.05 via DESeq2).

We utilized two forms of unbiased whole-transcriptome data dimensionality reduction, hierarchical clustering and principal component analysis (PCA). Both analysis methods showed separation predominantly by model type (Figure 2A,C). This suggests that shear accounted for less of the transcriptional variability between the samples than species and model cell type differences. Given the known species-specific differences at the BBB [57,58] and the differences in the stem-cell-derived models, it is perhaps unsurprising that the largest differences captured were between models and not between high and low shear. To directly assess the effects of shear on the transcriptome, we performed PCA on each model separately (Figure 2B), which showed a separation between low and high shear within each model based on PC1 and/or PC2, as expected.

Once it was evident that shear treatment drove a transcriptional change, we compared the transcriptional fold changes between low and high shear in each model via the DESeq2 package (via BioConductor version 3.15), which analyzes raw counts to compare two input conditions and accounts for the paired nature of our high- and low-shear samples. High versus low shear was compared in each model with hECs exhibiting the most differentially regulated genes (1253 Up, 1329 Down) and rBMECs (193 Up, 119 Down) exhibiting the fewest differentially-regulated genes (Table 1, Figure 2D). Of note, though many of the genes in hBMEC-like cells were regulated by shear, the magnitudes of the fold changes were more modest (<2-fold) compared to other models (Table 1). Complete lists of all gene expression TPM data (Supplemental File S3) and DESeq2 results (Supplemental File S4) are provided in the Supplementary Materials.

### 3.3. Canonical Shear Response Elements Are Upregulated in BBB Models

**Figure 3:**
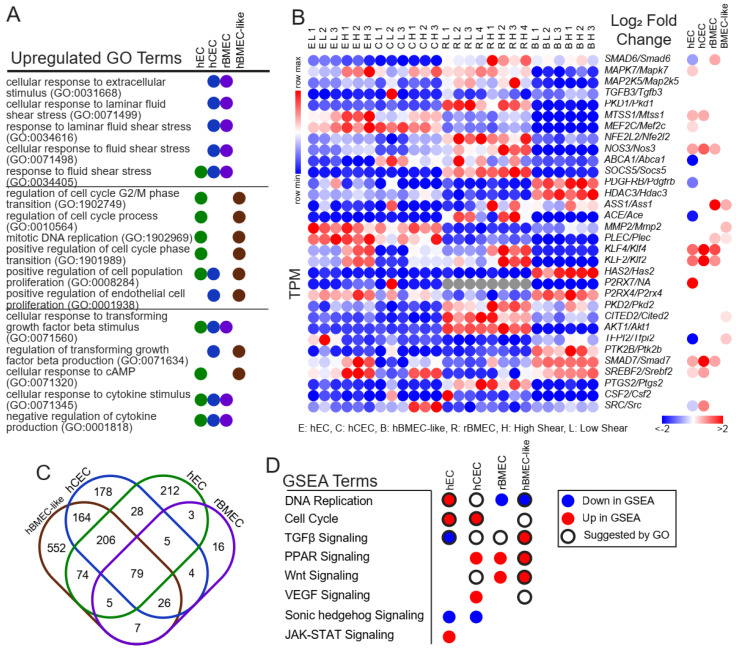
Pathway analysis and comparison between models. (**A**) Selected GO terms with an FDR < 0.05 returned from all differentially expressed genes in each model with a positive log_2_(fold change) > 0 and *p*adj < 0.05. The remaining GO terms are provided in Supplemental File S5. (**B**) Individual genes within the “response to fluid shear stress” (GO:0034405) gene set. Left is the TPM in each model replicate with relative expression. Right is the log_2_(fold change high versus low) as reported by DESeq2. All nonsignificant results are colorless (*p*adj > 0.05 via DESeq2). (**C**) Venn diagram of overlapping GO results between models. Numbers are the total GO terms. (**D**) Direction of the listed pathways if they were in the top twenty most enriched pathways according to GSEA analysis. Colors indicate the direction. Black rings indicate that GO terms related to this pathway were also enriched.

We next utilized several tools to identify shear-induced changes in gene expression. Statistically significantly (*p*adj < 0.05) and positively upregulated genes from DESeq2 were used to perform gene ontology (GO) analysis utilizing the biological process collection [43]. Every result is available in the Supplemental Information (Supplemental File S5). We observed terms the “response to fluid shear stress” (GO:0034405) or “response to laminar fluid shear stress” (GO:0071499) in all models except the hBMEC-like cells (Figure 3A), suggesting that the hECs, hCECs, and rBMECs respond in a canonical manner to shear flow. We generated a relative heat map of all genes in the “response to fluid shear stress” (GO:0034405) GO term, where we can see shear responsive elements, such as *KLF2/Klf2* and *KLF4/Klf4*, as well as the TGFβ signaling inhibitors *SMAD6/Smad6* and *SMAD7/Smad7* (Figure 3B). Since we used genes upregulated by shear in this analysis, genes downregulated by shear (e.g., *SMAD6* in hECs) were not included in the GO analysis list. Once it was clear that fluid flow was sufficient to induce a shear stress response in the models, we sought to identify signaling pathways that were affected by shear.

### 3.4. Endothelial Developmental and Signaling Pathways Are Transcriptionally Regulated by Shear

An analysis of the GO results identified shear-regulated terms relevant to key endothelial phenotypes and signaling pathways in each model (Figure 3A). However, GO does not take into account whether a gene is up- or down-regulated in a given pathway. Therefore, we also utilized Gene Set Enrichment Analysis (GSEA) [45] to compare a list of genes pre-ranked by the absolute magnitude of fold change and log of *p*adj (see Section 2) against those that would be expected to change in response to cataloged signaling pathways. GSEA terms responsive to shear included “DNA Replication” and “Cell Cycle”, along with developmental signaling pathways, such as TGFβ, Wnt, and VEGF signaling (Figure 3D). Interestingly, while some pathways were conserved amongst several models, such as PPAR and Wnt signaling, GSEA indicated that different models responded to shear in opposite directions for some pathways, including TGFβ signaling. A complete list of all GSEA results is provided in the supplement (Supplemental File S6).

We next coupled GO and GSEA analyses to infer shear effects on the different models. We initially compared which GO terms were upregulated in each model (Figure 3C). Among those shared between all samples were generic terms, such as the positive regulation of biological process (GO:0048518). Across all iPSC-derived models, but not rBMECs, we observed GO terms related to either DNA replication or cell proliferation, including “positive regulation of cell cycle phase transition” (GO1901989), “positive regulation of cell population proliferation” (GO:0008284), and “regulation of cell cycle G2/M phase transition” (GO:1902749), which suggests the transcriptional regulation of cell proliferation by shear (Figure 3A, Supplemental File S4). However, two GSEA gene sets, “Cell Proliferation” and “DNA Replication”, moved in opposite directions amongst the models (Figure 3D) with rBMECs and hBMEC-like cells both having reduced “DNA Replication” at high shear. By contrast, hECs and hCECs had the upregulation of “DNA Replication”, as well as “Cell Proliferation for hECs”, which suggests that the hECs and hCECs may be more proliferative under high shear.

GO terms associated with various signaling pathways were also identified upon shear flow (Figure 3A,D). TGFβ signaling, for example, was observed in all four cell types via GO analysis, with the terms “cellular response to TGFβ” (GO:0071560) and “regulation of TGFβ production” (GO:0071634). Similar to proliferation, GSEA results suggested different directions or a lack of response of TGFβ. hCECs and rBMECs showed no increase in TGFβ signaling, while hECs exhibited downregulated signaling, and hBMEC-like cells exhibited upregulated TGFβ signaling with shear. Some signaling pathways were identified, primarily via GSEA, in only the BBB models (hCECs, rBMECs, hBMEC-like cells) but not the generic hECs. In fact, several of these pathways are known to be associated with BBB property acquisition. The GSEA term for Wnt signaling, which is well known to be associated with BBB development [6,34,59], was upregulated in both rBMECs and hBMEC-like cells and identified in the GO analysis for the hCECs. Finally, the GSEA term for sonic hedgehog signaling, which has also been associated with BBB development [60], was decreased in both the hECs and hCECs. Overall, GO and GSEA analysis suggested that shear can affect key signaling cascades involved in both cell proliferation and BBB relevant developmental and signaling pathways.

### 3.5. A Subset of Key BBB Genes Is Shear-Responsive

Upon observing the upregulation of BBB-relevant developmental and signaling pathways via GSEA, we hypothesized that genes that confer key BBB properties may similarly be upregulated by shear. This led us to directly assess the impact of shear on a curated list of differentially expressed human BMEC genes that was assembled to include BBB tight junction and transcytosis components, as well as highly expressed solute carriers and efflux transporters, as determined from a meta-analysis of human brain scRNAseq data [34,61].

**Figure 4:**
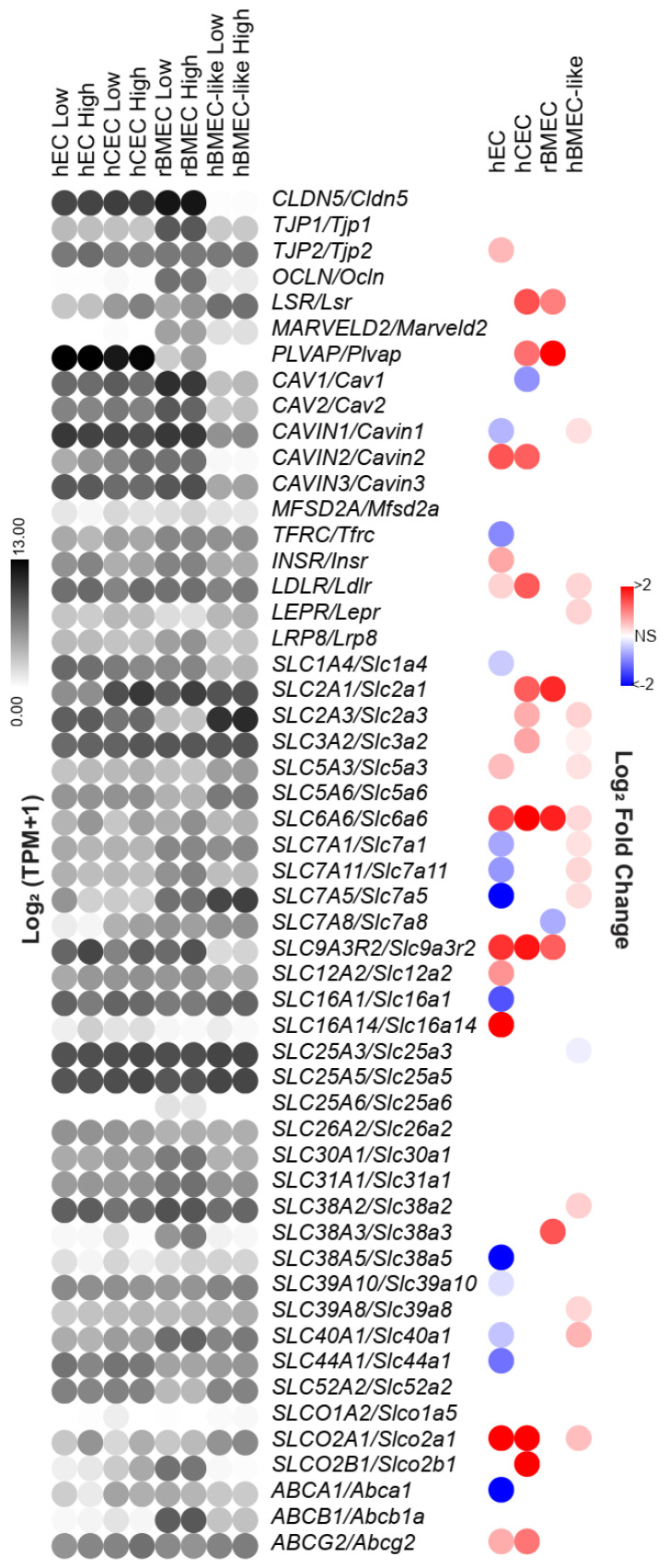
Shear regulation of BBB-associated genes. The gene list includes tight junction and vesicular transport genes and enriched *SLC* and *ABC* transporter genes identified as highly expressed in BMECs, identified in a compiled brain single-cell sequencing analysis. Since this list was compiled with respect to human BBB gene expression, rat gene names were converted utilizing the Homologene2 list. Left is the log_2_(TPM + 1) of the average expression in each model under low or high shear. Right is the log_2_(fold change) as a result of shear as reported by DESeq2. All nonsignificant results are colorless.

Several BBB-associated genes were upregulated by shear, and the shear-regulated genes differed between the models. For example, the expression of *SLC2A1/Slc2a1*, the gene encoding the GLUT1 glucose transporter which is highly expressed at the BBB [5], was increased by shear in both the rBMECs and hCECs but not in the hECs [34] (Figure 4). Similarly, *LSR/Lsr*, encoding lipolysis-stimulated lipoprotein receptor, which is typically localized at tri-cellular junctions, including at the BBB [62,63], was also increased in rBMECs and hCECs. Lastly, *CAV1/Cav1*, encoding caveolin 1, expected to be downregulated at the BBB, was slightly downregulated in hCECs under shear. Conversely, *PLVAP/Plvap*, known to be downregulated in the brain endothelium [64], was increased under shear in the hEC, hCEC, and rBMEC models. Interestingly, 15 BBB-associated genes were regulated by shear in hBMEC-like cells, with the bulk of these changes occurring in the transporter subset. Only *SLC6A6/Slc6a6,* which encodes a cation-dependent transporter, was regulated in a conserved manner across all models. Interestingly, the model lacking BBB character, hECs, had the most BBB-associated genes that were decreased by shear. Our analysis suggests that a subset of BBB-associated genes is regulated by shear stress and that this response is not conserved within the hEC model.

### 3.6. Identification of Potential Upstream Regulators That Could Drive Transcriptional Differences Between BBB Models and the hEC Model

**Figure 5:**
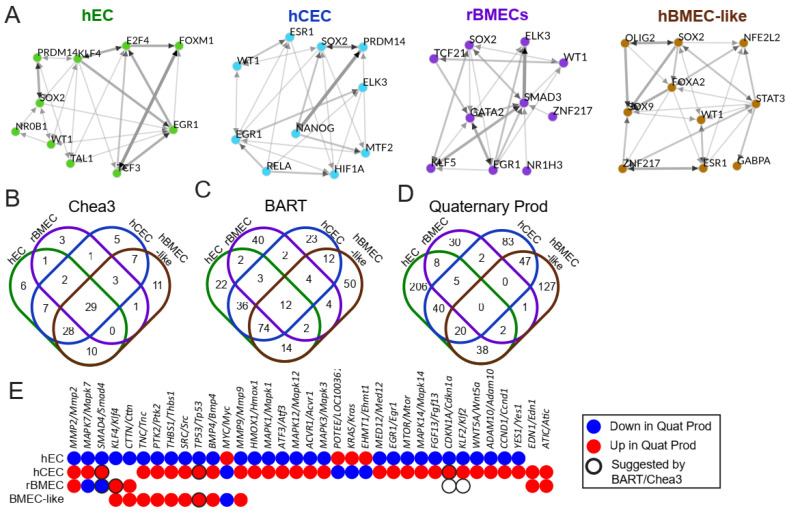
Upstream regulator analysis. (**A**) Top 10 enriched transcription factors from ChEA3 derived from a meta ChIP-seq in the “literature” library suggested from ChEA3 to account for upregulated genes between high and low shear in each model (with an FDR < 0.05). Graphs were generated using ChEA3 and represent the top 10 most statistically significant transcription factors that may account for differences between high and low shear in each model. The thickness and color of the arrow represent the number of overlapping downstream targets. (**B**) Venn diagrams demonstrating the total number of suggested upstream transcriptional regulators and overlaps between them. Results were generated using the “literature” library, and any transcription factor with an average TPM < 1 or FDR > 0.05 was removed. (**C**) BART ChIP-seq analysis results compared between the models. Any transcription factor with an average TPM < 1 or Irwin-Hall *p*-value > 0.05 was removed. (**D**) Quaternary Prod analysis results compared between the models. Any potential upstream regulator with an average TPM < 1 or *p*adj > 0.05 was removed. (**E**) Chart highlighting upstream regulators that were differentially regulated between hECs and either rBMECs or hCECs. Colors indicate the direction, and black rings represent transcription factors suggested by BART or ChEA3.

We hypothesized that the differential shear responses observed between the hEC and the BBB models could be driven by the differential expression of shear-responsive transcription factors or their associated machinery that results from their underlying BBB character. We employed multiple tools that have been developed to predict upstream regulators of transcriptional changes, including ChEA3 [47] and BARTweb [48] (Figure 5). Both utilize previously published ChIP-seq datasets and predict potential transcription factors that could account for any observed transcriptional changes. ChEA3 and BART analyses were performed with all genes differentially upregulated at high shear according to DESeq2 for each model. The transcription factors themselves do not need to be differentially expressed under high shear. However, we did limit our results to transcription factors with an FDR/Irwin-Hall *p*-value (as reported by ChEA3/BART respectively) of less than 0.05 and an average expression of at least 1 TPM to ensure that the transcription factor was expressed (Figure 5A–C). Similar to GO analysis, ChEA3 and BART do not account for the fold change. Therefore, we also utilized Quaternary Prod [65], which takes both fold change and *p*adj from DESeq2 into account while predicting potential upstream regulators. Further, and partially accounting for differences in outputs between the analysis methods, Quaternary Prod includes both transcription factors and other upstream regulators, like signaling proteins as potential driving factors (Figure 5D, Supplemental File S9). We then compared transcriptional regulators generated by each analysis package and identified those that were differentially regulated between hECs and either hCECs or rBMECs, to identify potential regulators of BBB-selective responses to shear (Figure S4).

Upstream regulators suggested by ChEA3, BART, or Quaternary Prod identified pathways known to be involved in BBB development and reinforced hits from the prior analysis (Figure S4). For example, *SMAD4* is involved in TGFβ superfamily signaling transduction, while *RXRA* and *RARG* are retinoic acid receptors (Figure S4B). This is consistent with GSEA results, which saw changes in both TGFβ and PPAR signaling in response to shear in some of the models (Figure 3D). Quaternary Prod returned two additional hits, *ATIC* and *EDN1,* which were elevated in hCECs and rBMECs and not identified in hECs (Figure 5D,E). *EDN1* encodes a precursor to endothelin-1, a signaling protein that drives vascular contraction, though interestingly, this regulator was not predicted to be involved in the hEC response to shear [66]. Next, we identified regulators with different directionality in response to shear in the BBB (hCEC and rBMEC) and non-BBB (hEC) models (Figure 5E). Of the 5 upstream regulators shared between these three models (Figure S4), 3 were differently regulated and of the 75 between hECs and either hCECs or rBMECs, 33 were differently regulated (Figure 5B–D). Among the differently regulated upstream regulators were *SMAD4* and the shear-responsive transcription factors *KLF2* and *KLF4* (Figure 5E and Figure S4).

### 3.7. Restoration of Shear Stress Is Not Sufficient to Prevent Dedifferentiation over Prolonged In Vitro Culture

**Figure 6:**
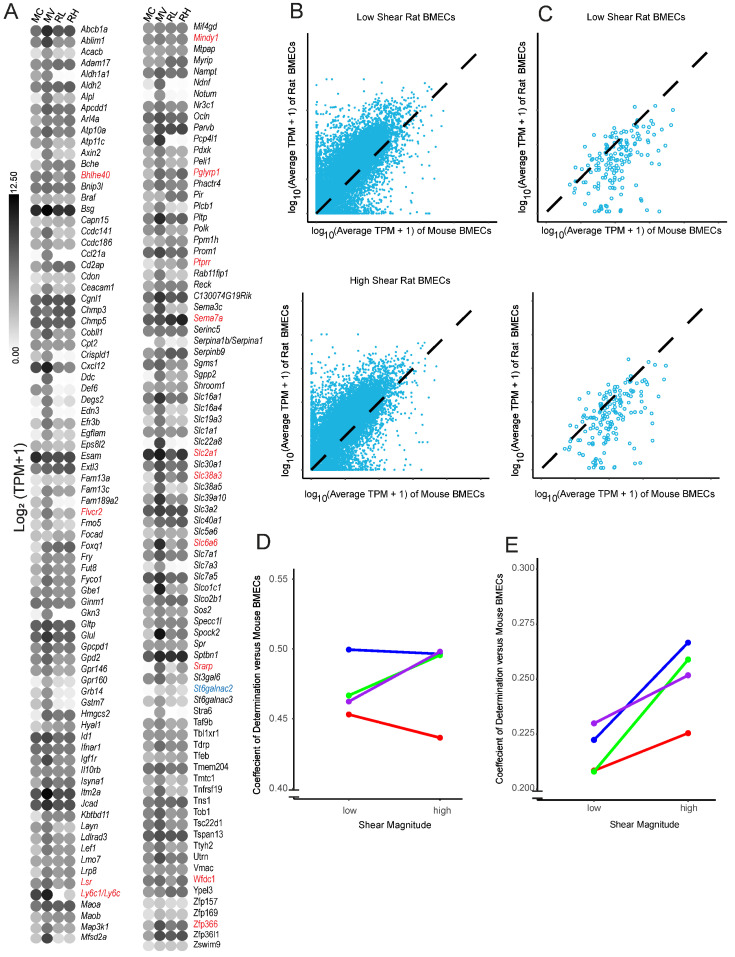
Comparison of rBMECs cultured with and without shear to acutely isolated mBMECs. (**A**) A list of BBB-relevant genes for which expression declined under long-term in vitro culture conditions [50] in mBMECs. The color of the dot represents the log_2_(average TPM + 1) from either our dataset or Sabbagh et al., 2020 [50]. Names are mouse genes. Cultured mBMECs (MC), acutely isolated mBMECs (MV), rBMECs under low shear (RL), and rBMECs under high shear (RH). Genes from this list that showed a statistically significant change via DESeq2 are in red font if upregulated and blue font if downregulated by shear in the current study. (**B**) Example plot of a linearity comparison between rBMECs under low or high shear and prior work with acutely isolated mBMECs. The TPM from rBMECs in high or low shear (y axis) versus the average TPM of the matched gene in acutely isolated mBMECs (x axis) is shown. Black dashed lines represent the theoretical perfect alignment. (**C**) Same linearity comparison as B, but with a limited dataset, including only those genes that were identified by Sabbagh et al. [50] as BBB downregulated in cultured mBMECs. (**D**) Coefficients of determination (R^2^) for whole transcriptomes from each linearity graph (B) for every rat isolation vs. acutely isolated mBMECs. Colors represent separate rBMEC isolations, and lines connect paired shear datapoints. No statistical significance was observed via a paired Student’s *t*-test. (**E**) Identical analysis to (**D**), except instead of assessing the whole-transcriptome alignment, only those BBB genes lost upon in vitro mBMEC culture are evaluated. *p* < 0.05 via paired Student’s *t*-test. Colors represent separate rBMEC isolations, and lines connect paired isolations.

Finally, we assessed if shear stress helped to maintain a BBB-like transcriptome in cultured rBMECs. It has long been known that BMECs dedifferentiate upon in vitro culture. Previous work [30,31] and some of the data presented above (Figure 4) suggest that some aspects of dedifferentiation could be reversed by the application of shear stress. A recent transcriptomic analysis of the impact of dedifferentiation in mouse BMECs (mBMECs) identified 164 genes associated with BBB and endothelial phenotypes that are downregulated in mBMECs as a consequence of in vitro culturing [50] (Figure 6A). To explore whether shear could substantially impact culture-induced dedifferentiation, we compared rBMECs under low (RL) and high (RH) shear to the aforementioned dataset, which compared acutely isolated mBMECs (MV) to cultured mBMECs (MC) [50]. Initially, we assessed whether high shear stress rescued the expression of any of the 164 BBB genes reportedly lost during long-term in vitro culture (Figure 6A). The rBMECs were cultured in vitro for a similar length of time to the mBMECs in culture, and several genes, such as *Acacb, Ccl21a/Ccl21*, *Notum*, and *Slc7a3*, exhibited reduced expression, similar to what was observed with the cultured mouse BMECs. We observed that 14 out of the 164 genes were expressed at a higher level in rBMECs under high shear, including *Slc2a1*. Only one gene, *St6galnac2*, was downregulated both by high shear and extended culture. To more quantitatively determine if the application of high shear drives a more in vivo-like transcriptome we calculated the coefficient of determination (R^2^), which functionally represents the goodness of fit of each rBMEC sample to the average TPM of acutely isolated mBMECs over the entire transcriptome (Figure 6B). However, when looking at the whole transcriptome, there was no statistically significant improvement in R^2^ (Figure 6D), suggesting that the application of high shear did not generally make the transcriptome of the rBMECs more similar to that of the in vivo mBMECs. We then limited the analysis to only those BBB genes listed in Figure 6A that are downregulated upon in vitro culture [50]. There was a small but statistically significant improvement in R^2^ (Figure 6C,E). Overall this suggests that high shear may positively impact some of the BBB genes reduced upon in vitro culture but not when considering the global transcriptome.

## 4. Discussion

Here, we performed bulk RNA sequencing on a naïve EC model and three models that have been used to represent various properties of the BBB. The BBB models were chosen to capture a broad swath of potential usages. rBMECs are a definitively specified primary cell model with BBB-relevant efflux activity, though previous work has identified de-differentiation that occurs during in vitro culture [24,38,50]. The recently reported hCECs express high levels of endothelial markers and several BBB transporters [34,35]; however, they lack physiological barrier properties and efflux transporter functions. Finally, hBMEC-like cells possess substantial tight junctions and efflux transport enabling the modeling of resistance of the BBB, but they express low levels of endothelial-specific genes and proteins, limiting their utility for studies regarding endothelial function and BBB development. This hBMEC-like model in particular has been utilized in microfluidic constructs to model the BBB in a physiologic mechanical environment [26,31,54,67,68,69,70]. Our analysis contributes to a better understanding of the transcriptional impact of shear on in vitro cellular BBB models in a head-to-head experimental comparison between primary rBMEC and iPSC-derived models. Our results demonstrate that the application of shear to in vitro BBB models does not provide a uniform cell response and that users of these models should consider shear responsiveness in their selection of models. While all models tested in this study exhibited shear-responsive gene transcription, shear was unable to restore significant in vivo-like characteristics to primary rBMECs and did not substantially induce BBB-like phenotypes in hPSC-derived cells. However, shear did induce a modest number of BBB-associated genes, suggesting that it can be a valuable tool for enhancing some BBB functionality in the models, depending on the application. For example, shear increased the expression of *SLC2A1/Slc2a1*, *SLC6A6/Slc6a6*, and *LSR/Lsr* in the hCEC and rBMEC models, suggesting that shear increases active transporter activity in these models, but these effects were not seen across all models tested. Thus, when choosing specific BBB models and whether or not to apply shear, researchers should carefully consider whether it influences the properties relevant for the desired application. Using the transcriptome data reported here, researchers could predict whether the application of shear will improve model performance to assess the BBB permeability of drug compounds, model the effects of disease on BBB phenotypes, and other applications.

To ensure that the geometry of the cell culture substrate and medium depletion effects did not impact the analysis of the effects of shear stress, we utilized low, but non-zero, shear stress applied to cells in the microfluidic chips as a comparative low-shear control. A low level of fluid flow ensured that the cells under low shear conditions (~0.5 dyne/cm^2^) did not suffer nutrient or oxygen depletion over the 72 h treatment [32,71]. While this low shear condition is below that typically required to induce shear-responsive gene expression and phenotypes in ECs [10,11,72], the culture conditions represent the closest comparative control for an evaluation of the impacts of physiological shear conditions.

Our analysis revealed hundreds of differentially regulated genes between high and low shear across the four models. hBMEC-like cells demonstrated the fewest genes that changed more than 2-fold in either direction (Table 1), perhaps as a result of the lower endothelial nature of these cells. rBMECs demonstrated the fewest differentially expressed genes overall, and this may be partially explained by their previous exposure to shear in vivo. Transcriptional network analysis using Quaternary Prod identified conserved networks enriched by shear in hCECs and rBMECs (e.g., *MMP2/Mmp2*, *EDN1/Edn1*, and *ATIC/Atic*) and in hBMEC-like cells and rBMECs (e.g., *KLF4/Klf4* and *CTTN/Cttn*). However, the hPSC-derived cells exhibited transcriptional network enrichment that was not found in the rBMECs, and these networks depended on the specific model (Figure 5). The basis of these differences is not clear and may be related to species specificity, a lack of complete BMEC specification in the hPSC-derived cells, and/or differences in the derivation and culture of the cells prior to application of shear. Our results also demonstrated that shear-regulated genes in hECs, hCECs, and rBMECs were associated with GO terms relevant to shear stress (Figure 3A), individual shear-responsive transcripts (Figure 3B), and pathways involved in the shear response via GSEA (Figure 3D). The hBMEC-like cells experienced minor increases in several shear-response elements according to GO, but did not return any statistically significant terms relevant to the shear response. Cell proliferation terms, shown to be reduced in endothelial cells under shear stress and in some BMEC models [8,10,11,73,74], were upregulated in hEC and hCEC GSEA results and downregulated in rBMECs (Figure 3D). Some of these differences may stem from the duration of shear treatment. Prior work, which demonstrated that immortalized BMECs and hBMEC-like cells do not align under shear, did observe, via video analysis, an increase in proliferation at 40 h, roughly half of our treatment duration. It is also possible that GSEA did not accurately assess the transcriptional readout, given that we used the 20 terms with the highest and lowest enrichment factors, which emphasizes the importance of utilizing multiple analysis packages, as well as follow up experiments [24,25]. Similarly, TGFβ signaling is known to increase in ECs under short-term (<24 h) shear application [15,16,75]. We observed GO terms relevant to TGFβ signaling in all four models, and GSEA suggested the downregulation of TGFβ signaling in the hECs, while Quaternary prod analysis suggested that SMAD4 and BMP4, both components of TGFβ superfamily signaling, were differentially regulated, between the hECs and BBB models as well as matching the downregulation predicted by GSEA for hECs. It is possible that compared to a 24 h shear duration, long-term shear application has led to feedback inhibition in our hECs. This is consistent with some of our signaling results, as well as Quaternary Prod results for *KLF2* and *KLF4*, which were both listed as downregulated upstream regulators in hECs (Figure 5C) despite being upregulated at the transcriptional level (Figure 3C) and well known to drive the endothelial shear response in the literature [21,22,23].

Interestingly, the expression of some BBB-associated genes increased under shear, including *LSR* and *SLC2A1* in rBMECs and hCECs. This suggests some reversal of the loss of BBB-relevant transcription under in vitro culture at the transcriptional level (Figure 4). Along these lines, we observed a slightly more in vivo-like rBMEC transcriptome after treatment with high shear, but only with regards to BBB genes, whereas across the global transcriptome, there was no measurable impact due to shear (Figure 6). However, by assessing the effects of shear on various BMEC and EC models after 72 h, we identified genes and pathways regulated by chronic shear. This time frame also limits the conclusions that we can make regarding acute, transient responses to shear. It is possible that over the short term, a different transcriptional response may occur [76].

## 5. Conclusions

Our study demonstrates that primary and hPSC-derived endothelial cells are shear-responsive, but that specific genes and pathways regulated by shear differ by model. Notably, genes regulating molecular transport respond to shear in a manner consistent with the BBB character in rBMEC and hCEC models. Our data provide a valuable resource to inform in vitro BBB model use, as well as the impacts of longer-term shear on these cell types that have previously been more significantly characterized under static conditions [1,12,41]. Given that shear-based BBB systems possess much higher experimental complexity, researchers seeking to deploy these models should use the provided datasets to consider the impacts that shear has on the expression of their gene(s) of interest. For instance, we found that shear stress alone only modestly rescued the BBB de-differentiation caused by long term culture, and therefore, the impacts of shear on BBB property induction in many cases may not outweigh the experimental challenges of implementation. These findings also motivate future work on elucidating the mechanisms by which various BMEC models respond to shear stress and how shear stress can influence BBB phenotypes in these models.

## Figures and Tables

**Table 1:** Summarized results of numbers of features differing between high- and low-shear conditions. The numbers represent features with both a *p*adj < 0.05 from DESeq2 and a fold change greater than 2.

Model	hEC	hCEC	rBMEC	hBMEC-like
**Upregulated**	1253	333	193	839
**Downregulated**	1329	110	119	567
**Upregulated (>2 fold)**	480	187	92	18
**Downregulated (>2 fold)**	558	81	42	11
**GO Hits (Up)**	611	689	144	1112
**GO Hits (Down)**	66	67	0	275
**ChEA3 Hits**	83	82	40	90
**BART Hits**	165	166	65	172
**Quaternary Prod**	331	208	49	241

## Data Availability

All raw sequencing data are available at GEO Accession Number GSE277559.

## Supplementary Materials

The following supporting information can be downloaded at: https://www.mdpi.com/article/10.3390/biom15020193/s1, Table S1: Antibodies utilized; Figure S1: Differentiation and rBMEC culture schemes; Figure S2: Microfluidic device setup; Figure S3: Minus Average (MA) plots for each of the models; Figure S4: Transcription factor analysis; File S1: RNAQCReport; File S2: Counts; File S3: TPM; File S4: DESeq2; File S5: GO; File S6: GSEA; File S7: ChEA3; File S8: BART; File S9: Quaternary Prod.
