# Supplementary material for: Transcriptional Responses of In Vitro Blood–Brain Barrier Models to Shear Stress
Source: Biomolecules. 2025 Jan 29;15(2):193. doi: 10.3390/biom15020193 (PMC11853657; doi:10.3390/biom15020193)
Supplement: Supplementary file 1 [file biomolecules-15-00193-s001.zip › biomolecules-3408463-supplementary.pdf]

Table S1 Antibodies utilized

**Target**	**Manufacturer**	**Cat. No.**	**Concentration**
Glut1	Invitrogen	MA1-37783	1:100 (ICC)
PECAM/CD31	Lab Vision	RB-10333 - P	1:100 (ICC)
Occludin	Invitrogen	33-1500	1:50 (ICC)
VE Cadherin/CD144	Santa Cruz	sc-52751	1:25 (ICC)
MRP1	Sigma Aldrich	MAB4100	1:50 (ICC)
Goat Anti-Mouse IgG (H+L) 488	Invitrogen	A11001	1:200 (ICC)
Goat Anti-Mouse IgG (H+L) 647	Invitrogen	A32728	1:200 (ICC)
Goat Anti-Rabbit IgG (H+L) 488	Invitrogen	A11008	1:200 (ICC)

4.9 Supplemental Figures

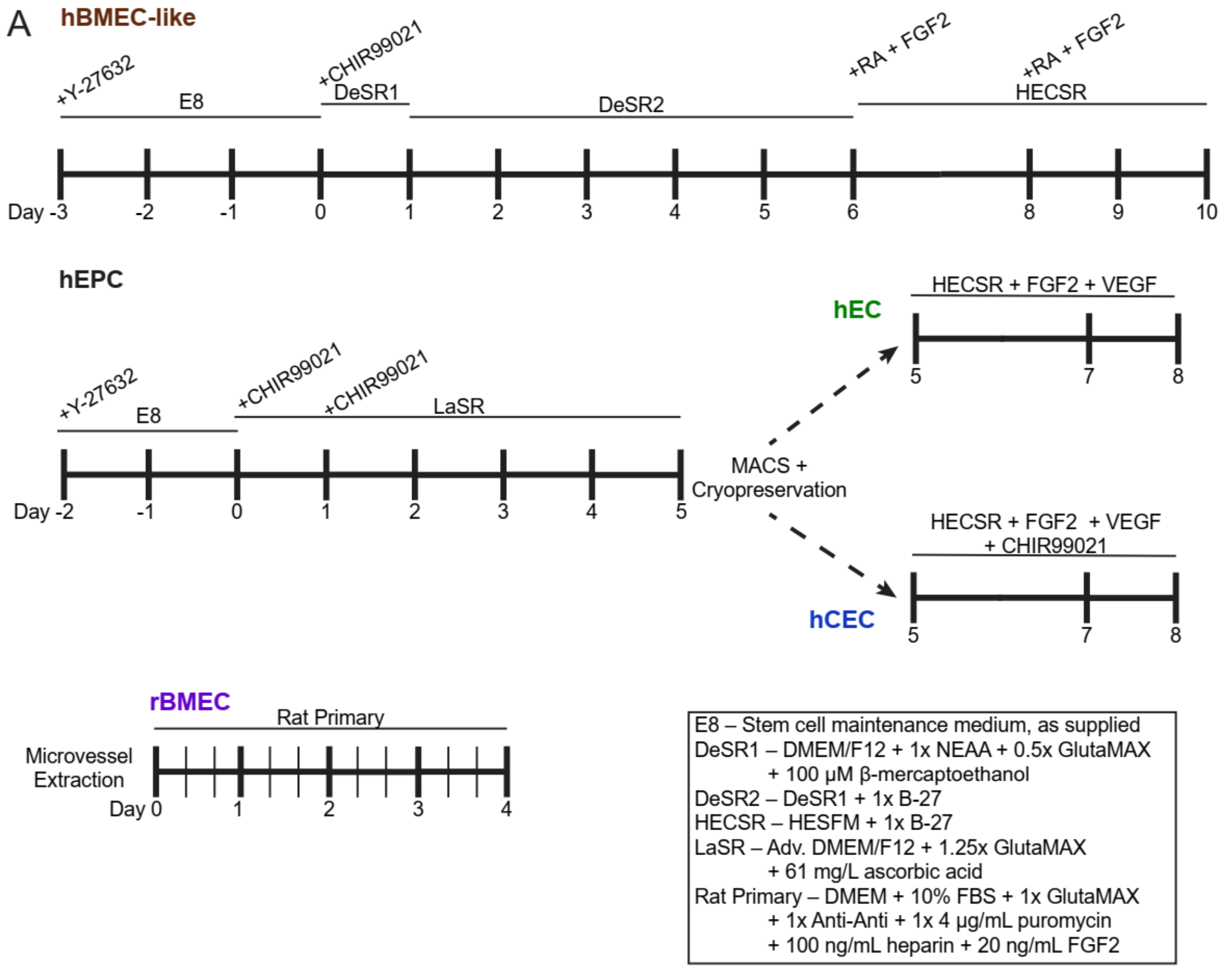

Figure S1 Differentiation and rBMEC culture schemes. Heavy vertical lines represent daily media changes, thinner vertical lines represent 8 hour media changes. Medium and supplements are provided above the timeline. Differentiated hBMEC-like, hEC and hCECs were replated into the microfluidic chips on the final day depicted. rBMECs were always in the chip due to passaging constraints.

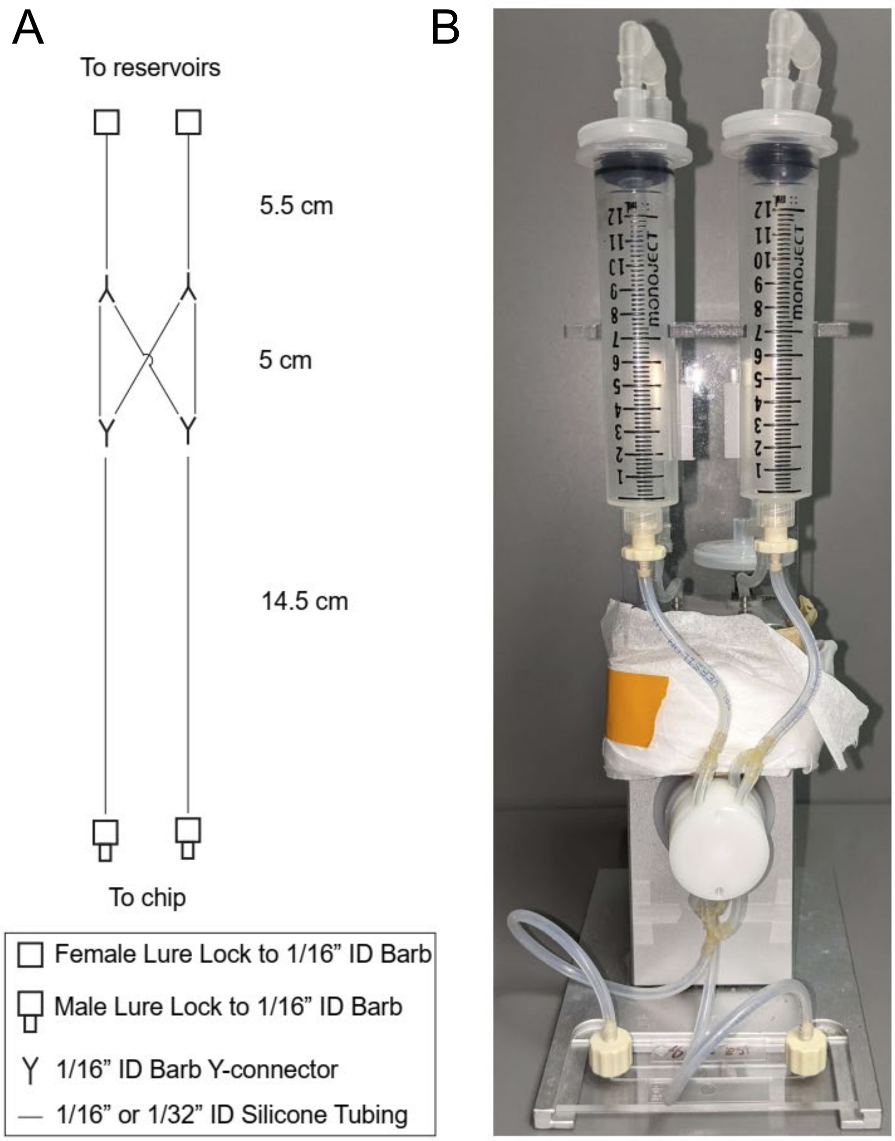

Figure S2 Microfluidic device setup. A) Diagram of tubing lengths and components utilized to operate the pump. B) Picture of a fully assembled pump with chip and reservoirs.

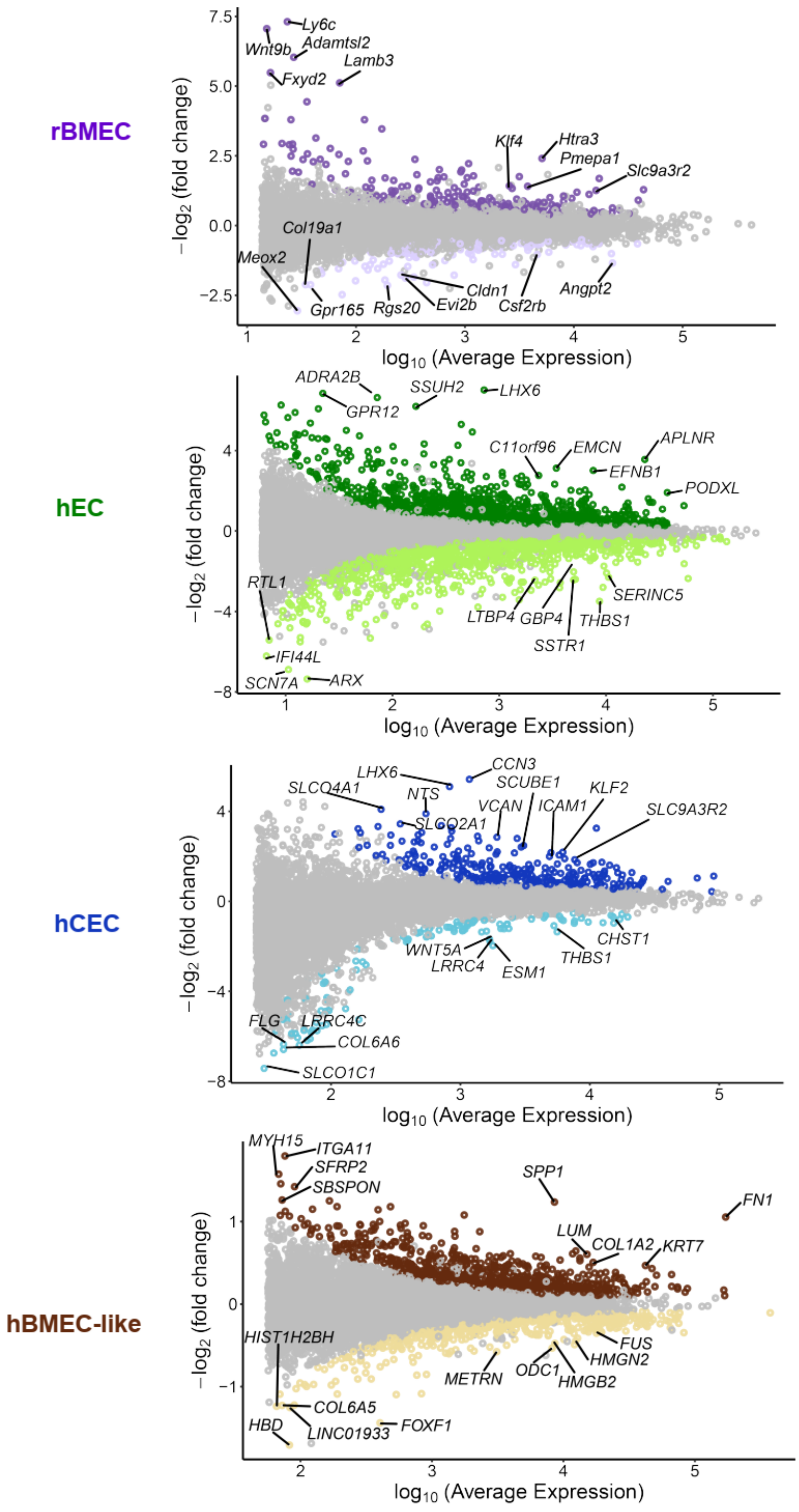

rBMEC
−log2 (fold change)
log10 (Average Expression)
Ly6c
Wnt9b
Adamtsl2
Lamb3
Fxyd2
Klf4
Htra3
Pmepa1
Slc9a3r2
Col19a1
Meox2
Gpr165
Rgs20
Evi2b
Cldn1
Csf2rb
Angpt2
hEC
−log2 (fold change)
log10 (Average Expression)
ADRA2B
SSUH2
LHX6
GPR12
C11orf96
EMCN
APLNR
EFNB1
PODXL
RTL1
SERINC5
LTBP4
GBP4
THBS1
SSTR1
IFI44L
SCN7A
ARX
hCEC
−log2 (fold change)
log10 (Average Expression)
CCN3
LHX6
SCUBE1
SLCO4A1
NTS
KLF2
VCAN
ICAM1
SLC9A3R2
SLCO2A1
CHST1
WNT5A
THBS1
LRRC4
ESM1
FLG
LRRC4C
COL6A6
SLCO1C1
hBMEC-like
−log2 (fold change)
log10 (Average Expression)
MYH15
ITGA11
SFRP2
SBSPON
SPP1
FN1
LUM
COL1A2
KRT7
HIST1H2BH
FUS
HMGN2
METRN
ODC1
HMGB2
COL6A5
HBD
FOXF1
LINC01933

Figure S3 Minus Average (MA) plots for each of the models. Labeled genes are the same as those marked on the volcano plots in Figure 2D and are the 5 genes with the smallest *p*adj and the 5 genes with the highest fold change.

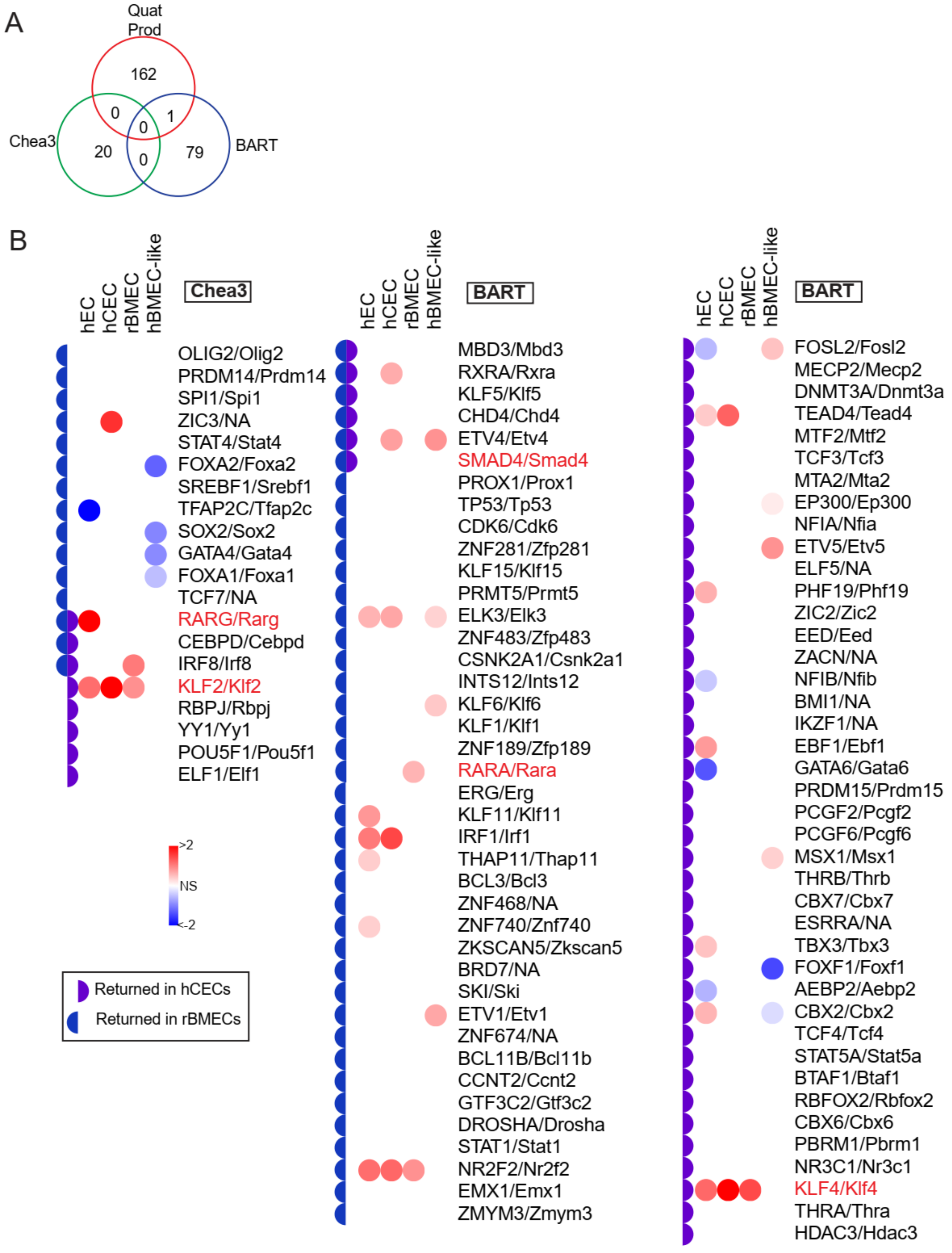
A
Quat
Prod
162
0
1
0
Chea3
20
0
79
BART
B
hEC
hCEC
rBMEC
hBMEC-like
Chea3
OLIG2/Olig2
PRDM14/Prdm14
SPI1/Spi1
ZIC3/NA
STAT4/Stat4
FOXA2/Foxa2
SREBF1/Srebf1
TFAP2C/Tfap2c
SOX2/Sox2
GATA4/Gata4
FOXA1/Foxa1
TCF7/NA
RARG/Rarg
CEBPD/Cebpd
IRF8/Irf8
KLF2/Klf2
RBPJ/Rbpj
YY1/Yy1
POU5F1/Pou5f1
ELF1/Elf1
>2
NS
<-2
Returned in hCECs
Returned in rBMECs
BART
MBD3/Mbd3
RXRA/Rxra
KLF5/Klf5
CHD4/Chd4
ETV4/Etv4
SMAD4/Smad4
PROX1/Prox1
TP53/Tp53
CDK6/Cdk6
ZNF281/Zfp281
KLF15/Klf15
PRMT5/Prmt5
ELK3/Elk3
ZNF483/Zfp483
CSNK2A1/Csnk2a1
INTS12/Ints12
KLF6/Klf6
KLF1/Klf1
ZNF189/Zfp189
RARA/Rara
ERG/Erg
KLF11/Klf11
IRF1/Irf1
THAP11/Thap11
BCL3/Bcl3
ZNF468/NA
ZNF740/Znf740
ZKSCAN5/Zkscan5
BRD7/NA
SKI/Ski
ETV1/Etv1
ZNF674/NA
BCL11B/Bcl11b
CCNT2/Ccnt2
GTF3C2/Gtf3c2
DROSHA/Drosha
STAT1/Stat1
NR2F2/Nr2f2
EMX1/Emx1
ZMYM3/Zmym3
BART
FOSL2/Fosl2
MECP2/Mecp2
DNMT3A/Dnmt3a
TEAD4/Tead4
MTF2/Mtf2
TCF3/Tcf3
MTA2/Mta2
EP300/Ep300
NFIA/Nfia
ETV5/Etv5
ELF5/NA
PHF19/Phf19
ZIC2/Zic2
EED/Eed
ZACN/NA
NFIB/Nfib
BMI1/NA
IKZF1/NA
EBF1/Ebf1
GATA6/Gata6
PRDM15/Prdm15
PCGF2/Pcgf2
PCGF6/Pcgf6
MSX1/Msx1
THRB/Thrb
CBX7/Cbx7
ESRRA/NA
TBX3/Tbx3
FOXF1/Foxf1
AEBP2/Aebp2
CBX2/Cbx2
TCF4/Tcf4
STAT5A/Stat5a
BTAF1/Btaf1
RBFOX2/Rbfox2
CBX6/Cbx6
PBRM1/Pbrm1
NR3C1/Nr3c1
KLF4/Klf4
THRA/Thra
HDAC3/Hdac3

Figure S4 Transcription factor analysis. A) Venn diagram comparing suggested upstream transcriptional regulators returned by the three methods utilized. Results include only those hits with TPM > 1, statistical significance, and that are present in either hCECs or rBMECs, but not hECs. B) Lists of all suggested upstream transcriptional regulators by either Chea3 or BART with an average TPM > 1 and FDR/Irwin-Hall p value < 0.05. All hits shown were selected due to presence in either rBMECs or hCECs. Red to blue colored dots represent $\log_2$(fold change) as reported by DESeq2. Non-statistically significant results were colored white.
